# A Novel Strategy to Increase Identification of African-Born People With Chronic Hepatitis B Virus Infection in the Chicago Metropolitan Area, 2012–2014

**DOI:** 10.5888/pcd13.160162

**Published:** 2016-09-01

**Authors:** Edwin Chandrasekar, Sharon Song, Matthew Johnson, Aaron M. Harris, Gary I. Kaufman, David Freedman, Michael T. Quinn, Karen E. Kim

**Affiliations:** Author Affiliations: Sharon Song, Matthew Johnson, Asian Health Coalition, Chicago, Illinois; Aaron M. Harris, Division of Viral Hepatitis, Centers for Disease Control and Prevention, Atlanta, Georgia; Gary I. Kaufman, Sinai Health System, Mount Sinai Hospital Touhy Health Center, Chicago, Illinois; David Freedman, Heartland Health Centers, Chicago, Illinois; Michael T. Quinn, Karen E. Kim, University of Chicago, Division of the Biological Sciences and Office of Community Engagement and Cancer Disparities, Chicago, Illinois.

## Abstract

**Introduction:**

Most research on hepatitis B virus (HBV) infection in the United States is limited to Asian populations, despite an equally high prevalence among African immigrants. The purpose of this study was to determine testing and detection rates of HBV infection among African-born people residing in the Chicago metropolitan area.

**Methods:**

A hepatitis education and prevention program was developed in collaboration with academic, clinical, and community partners for immigrant and refugee populations at risk for HBV infection. Community health workers implemented chain referral sampling, a novel strategy for recruiting hard-to-reach participants, targeting African-born participants. Participants were tested in both clinical and nonclinical settings. To assess infection status, blood samples were obtained for hepatitis B surface antigen (HBsAg), core antibody, and surface antibody testing. Demographic information was collected on age, sex, health insurance status, country of origin, and years residing in the United States. Participants were notified of testing results, and HBsAg-positive participants were referred for follow-up medical care.

**Results:**

Of 1,000 African-born people who received education, 445 (45%) agreed to participate in HBV screening. There were 386 (87%) participants tested in clinical and 59 (13%) tested in nonclinical sites. Compared with participants who were tested in clinical settings, participants tested in nonclinical settings were older, were less likely to have health insurance, and had lived in the United States longer (*P* < .005 for each). Of these, most were from the Democratic Republic of the Congo (14%), Nigeria (13%), Ghana (11%), Somalia (11%), or Ethiopia (10%). There were 35 (8%) HBsAg-positive people, 37% had evidence of past infection, and 29% were immune.

**Conclusions:**

Chain referral sampling identified many at-risk African-born people with chronic HBV infection. The large proportion of HBsAg-positive people in this sample reinforces the need for health promotion programs that are culturally appropriate and community-driven.

## Introduction

Infection with hepatitis B virus (HBV) is life threatening and may lead to acute and chronic liver diseases (1) including cancer and cirrhosis (2). Approximately 248 million people are estimated to be infected worldwide (3,4), and 686,000 annual deaths are associated with the disease and its complications (5). The highest burden is in Asian and African countries (6). Foreign-born Africans are a fast-growing immigrant group in the United States. During the past 3 decades, migration has increased from 200,000 to 1.7 million (7,8). According to the 2010 Census, the Chicago metropolitan area is home to approximately 50,000 African-born people, which is more than double the number counted in the 2000 Census (9).

Foreign-born people who enter the United States as refugees are granted asylum and undergo a comprehensive health assessment that includes screening for immunity to HBV infection. The medical evaluation of immigrants applying for permanent residency varies and does not incorporate testing for chronic HBV infection (10). An estimated 54,000 people with chronic HBV infection immigrate to the United States annually (8).

The health status of African-born people remains largely unexamined. Public health reports have focused on infectious diseases, including HIV and tuberculosis, and some studies have compared African immigrants with African Americans (11). As African-born people continue to migrate to the United States from diverse countries, surveillance of a broad range of chronic diseases and infections will be important, and health care practices will need to be aligned accordingly. An estimated 29% of people with chronic HBV infection in the United States are African-born (12). A cross-sectional chart review of African immigrants attending an urban clinic in Minnesota indicated that 14% had evidence of HBV infection (13). The prevalence of HBV infection among African immigrants in Europe is similarly high (14,15).

Most research on HBV infection in the United States is limited to Asian populations, despite an equally high prevalence among African immigrants (8). Little is known about the prevalence of chronic HBV infection among African-born people residing in the Chicago area. Improving the identification and public health management of immigrants with HBV infection is crucial to eliminating transmission and reducing the incidence of HBV-related liver diseases (16). The aims of this study were to use a novel strategy of chain referral sampling to conduct HBV testing and describe the prevalence of chronic HBV infection among a sample of people born in Africa and now residing in the Chicago metropolitan area.

## Methods

African-born community members were recruited to participate in the screening program through informational flyers and posters displayed at well-attended venues, including temples, grocery stores, and restaurants, and by word-of-mouth invitation. Participants received education on HBV infection and were offered hepatitis B virus testing at one of several community-based health clinics.

### Procedures

Enrollment into the program occurred in 3 waves. We first enlisted clientele from community-based agencies, including church groups, federally qualified health care clinics, and a refugee clinic affiliated with a multihospital health care organization. African-born adults were then selected through chain-referral sampling, an approach that has been used extensively in observational research to recruit otherwise hard-to-reach individuals (17). The method relies on natural social networks and integrates a series of participant-informed referrals into “chain links” so that the resulting sample closely resembles the population of interest. Finally, participants who attended the educational sessions on hepatitis B were offered complimentary testing for HBV infection, which included hepatitis B surface antigen (HBsAg) as well as hepatitis B surface antibody (anti-HBs), total hepatitis B core antibody (anti-HBc), or both.

Test results were categorized according to the following definitions: chronic infection (HBsAg+, anti-HBc+, anti-HBs−), past infection (HBsAg−, anti-HBc+, anti-HBs+ or −), immune (HBsAg−, anti-HBc−, anti-HBs+), and susceptible (HBsAg−, anti-HBc−, anti-HBs−). Specimens were sent to Quest Laboratories for testing. Participants who tested negative for HBV infection received language-concordant notification letters. Participants with positive test results were notified via telephone and a letter; they were provided post-test counseling and were referred to a health clinic or community-based organization. Community health workers assisted participants in making appointments with local health care providers. Immune participants were informed that no further follow-up was required. People identified as being susceptible to HBV infection received counseling and were offered hepatitis B vaccination. The screenings were performed in clinical and nonclinical settings from December 2012 through December 2014.

The clinic settings included community and federally qualified health centers, charitable organizations, solo and small group physician practices, and local hospitals. Physicians briefly educated participants about hepatitis B and offered a free blood test. Consent for the blood draws was obtained from participants, and demographic and risk factor data were collected. Results of the screenings were provided directly to participants.

Nonclinical settings consisted of health fairs and other events hosted by community and faith-based organizations. Screenings followed educational workshops delivered in the target languages of participants or interpreted by trained bilingual community health workers. Community health workers assisted participants with the consent forms, and volunteer nurses and phlebotomists performed the blood draws.

### Data analysis

All data were analyzed using SPSS version 21 (IBM Corporation). Participants were characterized demographically on the basis of sex, race, age, health insurance status, years living in the United States, country of birth, type of setting in which they were seen, and HBV infection status. Differences between the clinical and nonclinical settings were evaluated using the χ^2^ test, and significance was set at *P* < .05. The University of Chicago Medicine’s institutional review board approved the study protocol.

## Results

Approximately 3,000 Asian- or African-born immigrants with limited English skills received education on HBV infection. Of those, 1,000 were African, and 445 (45%) African-born participants underwent HBsAg testing in several health and community-based clinics (Table 1). Most participants were black (93%), median age was 33 years, and most were residents of the United States for less than 10 years (71%). The proportion of uninsured participants in nonclinical settings was significantly higher than the proportion in clinical settings (54% vs 13%; *P* = .005). Participants tested in nonclinical settings were older than those tested in clinical settings (*P* < .001), and most had resided in the United States for more than 10 years, whereas most of those tested in clinical settings had lived in the United States for fewer than 10 years (*P* < .001) (Table 1).

**Table 1:** Demographic Characteristics and Testing Results of African-Born Persons Recruited Through a Chain Referral Sampling Strategy, Chicago Metropolitan Area, 2012–2014

Variable	Number (%) (N = 445)	Clinical Screening Location (%) (N = 386)	Nonclinical Screening Location (%) (N = 59)	*P* Value
**Sex**				
Male	219 (49)	186 (48)	33 (56)	.33
Female	226 (51)	200 (52)	26 (44)	
**Race**				
Black	411 (93)	353 (91)	58 (98)	<.004
White	11 (2)	11 (3)	0	
Other	12 (3)	11 (3)	1 (2)	
Not indicated	11 (2)	11 (3)	0	
**Age, y**				
<18	90 (20)	90 (23)	0	<.001
18–39	190 (43)	174 (45)	16 (27)	
40–64	156 (35)	115 (30)	41 (70)	
≥65	9 (2)	7 (2)	2 (3)	
**Health insurance status**				
Yes	354 (80)	330 (86)	24 (41)	.005
No	83 (19)	51 (13)	32 (54)	
Unknown	8 (2)	5 (1)	3 (5)	
**Residency in United States, y**				
<10	310 (70)	300 (78)	10 (17)	<.001
10–19	29 (6)	0	29 (49)	
20–29	14 (3)	0	14 (24)	
Unknown	92 (21)	86 (22)	6 (10)	

Thirty-five (8%) of 445 participants receiving HBV screening were HBsAg positive. Of 194 with complete serological data for all 3 tests, 72 (37%) had evidence of past infection, 57 (29%) were immune, and 50 (26%) were susceptible. There were 231 participants who had only HBsAg results available; 60 (13.5%) participants received at least 1 dose of hepatitis B vaccine. Participants were from 33 African countries, with the largest proportions from Democratic Republic of the Congo (14%), Nigeria (13%), Ghana (11%), Somalia (11%), and Ethiopia (10%). HBsAg and anti-HBc testing results by country of origin are shown in Table 2.

**Table 2:** Proportion of Hepatitis B Surface Antigen and Potential Exposure Based on Hepatitis B Core Antibody Positivity Among African-Born Persons by Country of Origin, Chicago Metropolitan Area, 2012–2014

Country of Origin	HBsAg and Anti-HBc Results		
	HBsAg Positive, No. (%)	Anti-HBc Positive^a^, No. (%)	Total No. Tested
Algeria	0	0	2
Benin	0	0	1
Cameroon	0	0	4
Central Africa Republic	3 (43)	0	7
Chad	0	0	6
Democratic Republic of the Congo	3 (5)	21 (11)	62
Cote d’Ivoire (Ivory Coast)	1 (33)	2 (1)	3
Djibouti	2 (50)	0	4
Egypt	0	2 (1)	3
Eritrea	0	0	6
Ethiopia	2 (4.5)	2 (1)	44
Ghana	4 (8)	1 (.05)	49
Guinea	0	0	1
Kenya	0	6 (3)	11
Liberia	1 (33)	0	3
Libyan Arab Jamah	0	2 (1)	4
Malawi	0	0	2
Mauritania	0	1 (.05)	2
Morocco	0	0	4
Mozambique	0	0	3
Namibia	0	1 (.05)	5
Niger	0	0	2
Nigeria	4 (7)	4 (2)	58
Rwanda	1 (4)	1 (.05)	26
Sierra Leone	1 (50)	1 (.05)	2
Somalia	7 (15)	13 (7)	47
Sudan	5 (25)	4 (2)	20
Syria	0	0	2
Tanzania	0	1 (.05)	2
Togo	0	1 (.05)	8
Uganda	0	6 (3)	13
Zaire	1 (3)	0	34
Zimbabwe	0	0	2
Other Africa^b^	0	3 (1.5)	3
**Totals**	35 (8)	72 (37)	445

Abbreviations: anti-HBc, hepatitis B core antibody; anti-HBs, hepatitis B surface antibody; HBsAG, hepatitis B surface antigen.

a 194 participants had serological data for all 3 tests (HBsAg, anti-HBc, anti-HBs).

b Three participants indicated “Africa” rather than a specific country.

## Discussion

Results from this study demonstrate the utility of chain referral sampling to engage African-born people in educational programs and screenings for HBV infection. We tested 45% of the target population and identified 8% with chronic HBV infection.

The strategy leveraged community health workers, in conjunction with peer and faith-based leaders, who were in the position to engage a hard-to-reach population. Community health workers are instrumental in engaging underserved populations (18). They have effectively intervened with populations experiencing chronic conditions such as diabetes and HIV (19,20) and have assisted in implementing culturally tailored programs to reduce health disparities in ethnically diverse populations (21). We reached a population who needed services but were limited by access and language barriers. Chain referral sampling helped to overcome some challenges of limited English language comprehension, illiteracy, and mistrust. Our reach included nearly 20% of people with no insurance and many recent immigrants with fewer than 10 years of residency in the United States. Additionally, the strategy led to identification of people susceptible to HBV infection and facilitated hepatitis B vaccination. Building on the links that the community-based organizations and their clientele comprised, the referral chains lengthened and the sampling pool widened as interest in the program broadened to a point at which community members themselves became engaged and even recruited others to participate. Establishing visibility in African immigrant communities and generating interest in future workshop cycles further lengthened the social network links.

Similar success with an HBV testing program among immigrant populations was identified in Europe using a similar community-based approach (22). Stornaiuolo et al (23) examined the impact of proactive participant recruitment in an Italian community that was home to 5 million immigrants. They used the mobile unit of a health and family counseling center dedicated to providing HIV screenings of hard-to-reach participants and compared the results to those obtained in their outpatient health clinic alone. The number of HIV positive people identified over a 6-year period was significantly higher using the mobile unit than without it. Similar to our study, intervention directly in the community led to screening and detection rates that we assert are difficult to achieve through clinic-based practice alone.

There were many lessons learned when using a chain referral sampling strategy to test African-born people for HBV infection. Factors such as lack of health insurance, limited English proficiency, ineligibility for public assistance programs, cost of health services, lack of medical interpretation services, and the absence of legal documentation commonly are barriers to obtaining health care services (24). In this study, reluctance to self-identify as a recent immigrant due to fear of reprisal probably compounded the utilization challenges as well as the stigma associated with the disease. The recruitment strategy helped to mitigate this distrust because of the natural social networks that contextualized participants’ involvement. Recommendation for HBV testing by respected fellow members of the community promoted buy-in. Some community-based organizations may have experienced communication challenges when assisting participants with low literacy even when the explanations of the HBV testing protocol and demographic questionnaires were translated into their spoken languages. Future studies should standardize recruitment processes at screening events and the role of community health workers, particularly those with linguistic and cultural competence.

There are at least 3 limitations to this study. First, the large proportion of missing data may have led to misclassification of HBV infection status. Second, it was not possible to screen the entire population for HBV infection and follow-up with people found to be infected, both of which are necessary for reducing disease prevalence. Cultural barriers including denial and social stigma, as well as concerns about the cost of treatment if tested positive, tended to perpetuate racial/ethnic disparities in hepatitis B disease despite the involvement of peers. Third, we were unable to assess linkage to care for people who tested HBsAg positive. Strategies that increase linkage to care for African-born people that test HBsAg positive are needed.

Despite these limitations, we were able to use the chain referral strategy to implement HBV testing and identify many African-born people with chronic HBV infection. The results indicate that there is a need for health promotion programs that are culturally appropriate and community-driven. Although the US Department of Health and Human Services has made substantial efforts to improve hepatitis B surveillance practices, including Centers for Disease Control and Prevention–led initiatives to systematically gather HBV-related health information on racial/ethnic minorities (25,26), this article highlights the need to develop HBV-related education, screening, and linkage-to-care strategies that target African-born people.
